# Addressing mental health, misinformation, & religious tensions among South Asian students across California higher education during the COVID-19 pandemic: A qualitative research study

**DOI:** 10.1016/j.heliyon.2023.e16396

**Published:** 2023-05-19

**Authors:** Zobia Quraishi

**Affiliations:** Department of Public Policy, University of California, Berkeley, CA, USA

**Keywords:** Mental health, Students, Health, COVID-19, Distance learning, Misinformation, Tolerance, South Asian

## Abstract

**Background:**

South Asian Americans comprise one of the fastest-growing ethnic groups in the US. Nevertheless, the scientific literature on the experiences of South Asian students is lacking, where often studies focused on Asian Americans exclude South Asians altogether. South Asian students have unique experiences in California higher education, often having to cope with high pressure to perform academically and various social responsibilities associated with being first-, second-, or third-generation American.

Many South Asian countries have been more severely affected by COVID-19 due to the density of people living in close proximity to one another. Students of South Asian origin worry for their families overseas, especially as the COVID-19 pandemic has impacted populations there to a more considerable extent.

**Methodology:**

This was a qualitative research study conducted using in-depth interviews with 25 students who identified as South Asian and were currently enrolled in higher education in California at the time they were interviewed. Ads were placed on social media networks, such as Facebook and Twitter, and participation in the study was voluntary. Students were chosen randomly to avoid bias. Study participants described their experiences navigating higher education throughout the ongoing COVID-19 pandemic in relation to their social, economic, cultural, and political spheres. Interviews were mainly conducted through Zoom, some through emails or phone calls. All participants will remain anonymous to protect the identities of students.

This project sought to understand the experiences of South Asian students as they navigate the new normal amidst a global crisis. Qualitative responses were indexed by themes, with South Asian students reporting a relationship between the pandemic and worsened academic experiences, issues of exacerbated mental health, the spread of misinformation, and increased religious tensions.

This research reflects pragmatism, and the findings of this paper are coupled with suggested recommendations to ameliorate pandemic related issues for future South Asian students. The project was conducted through both inductive and qualitative approaches. The research strategy is action research, and the research was meant to shine a light on issues South Asian students face and help university administrations understand how to better support students during a pandemic. The time horizon is cross-sectional. The research focuses on COVID-19 in relation to South Asian students’ performance and health.

At 25 participants, data saturation was reached. Further participants were not needed as the qualitative research data was sufficient to draw conclusions from. The research was deemed to pose no risk to students; therefore, approval from an ethical committee was not sought. All participants signed consent forms in order to participate.

Follow-up interviews were conducted to address students’ experiences with family in India experiencing the B.167.2 variant and the transition back to in-person learning following virtual schooling.

**Conclusions/significance:**

South Asian students in higher education bear a unique set of responsibilities in addition to the already rigorous coursework associated with college. However, the stresses and health of South Asian students often go understudied or overlooked. This research serves to begin to fill in the gaps in literature of South Asian experiences in higher education.

The pandemic posed additional challenges for students, who lost access to their campus communities with university education going virtual, and many South Asian students had to move back home. Through this qualitative research study analyzing the experiences of South Asians enrolled in California higher education, it can be concluded that there is a relationship between the COVID-19 pandemic and students’ academic performance, mental health, as well as an increase in the amount of misinformation spread regarding COVID-19 public safety guidelines.

## 1 Introduction

Beginning in late 2019, the COVID-19 pandemic has devastated global society, infecting people of all ages and disrupting everyday life. COVID-19 is a respiratory disease with a range of effects on human health. The disease was found to have been caused by a novel coronavirus similar in structure to the virus that causes severe acute respiratory syndrome (SARS) [1]. Symptoms mainly include fever, cough, and shortness of breath. Others suffer from more severe symptoms such as loss of smell, loss of taste, nausea, diarrhea, arrhythmias, heart attacks, damaged kidneys or livers, headaches, blood clots, rashes, swelling, or strokes [2]. While most people who contracted COVID-19 were mildly affected, the disease can also lead to severe illness and, in worst-case scenarios, death [3]. The human-to-human transmission of COVID-19 was high, with cases increasing exponentially [4]. The implications of the global COVID-19 pandemic were vast. Financial markets were impacted on a global scale, and lockdowns led to a disruption in the supply chain [5]. In the US, economies fell, and the Coronavirus Aid, Relief, and Economic Security (CARES) Act passed to help alleviate the impact COVID-19 has had [6]. The financial hit American colleges and universities took due to the pandemic is estimated to be over $120 billion [7]. Students felt this loss in relation to their education, sacrificing the traditional learning experience for a virtual one.

The new reality associated with COVID-19 has altered education, with schools closing and most universities switching to distance learning when the pandemic initially hit. All across colleges in the US, college students had to adjust to a college experience they had not expected, as they were asked to leave their campus housing, adapt to virtual platforms, and continue to pay college tuition despite the financial downturn. Students were tasked with learning virtually as opposed to in the classroom for over a year at most universities, leading to Zoom fatigue and disengagement.

This project sought to illustrate how COVID-19 has changed the experiences of South Asian students enrolled in higher education in California and their extended families as they navigate furthering their education in an academic setting that is entirely different from normal. The interviews conducted through this study illustrate the narratives of South Asian students and their experiences in higher education during the pandemic.

## 2 Results & discussion

The COVID-19 pandemic has brought to light some of the issues South Asian students have always faced but now heightened as a result of the pandemic.

### 2.1 Limitations of this study

It is important to view this paper's findings considering its limitations. The research was conducted over the period of three years; therefore, not all nuances of the COVID-19 pandemic throughout this timeframe are captured within the findings of the paper. The majority of this research focuses on when the pandemic initially hit, mainly the three academic semesters (and two summer semesters) classes first went online.

There is a lack of previous research done on the impact of a pandemic on South Asian students and methods to mitigate negative impacts of COVID-19 for South Asian families. Therefore, while this research offers insight on a topic not thoroughly covered in research, the lack of previous research to reference limited the scope of the study.

Because many of the participants found the opportunity to participate in this research study through social media advertisements, there is a selection bias of individuals who use and consume media, who may be aware of the effects of COVID-19 through their social media use.

Discussion on how to mitigate these limitations for future research is included under the future guidance & recommendations for policy and research section of this paper.

### 2.2 Academics

School systems faced unprecedented challenges as the global pandemic forced instruction to go online beginning in the spring 2020 semester. Students and teachers alike navigated transitioning curricula online, test-taking, and making the most out of a less-than-ideal situation. Some elected not to continue with their pursuit of higher education due to circumstances surrounding the COVID-19 pandemic, and this trend was more common for students of lower-income backgrounds [8].

Students enrolled in college are facing a large amount of stress during the pandemic and do not have the same capacity to perform. A participant of Pakistani-American descent reflects on being caught off-guard by schooling going online, noting distractions from his younger sibling at home.

“[The transition to virtual learning] definitely has been very hard. I paid for in-person learning. It’s hard to gather myself around doing online school. I do a lot better in-person than I do online … Since school is being cancelled, my sister is online now. She’s been bouncing around the walls. She’s running around.” –Chand

When the pandemic started, students were unfamiliar with the online learning scheme, with more than half of college students having never taken a class online [9]. Maladaptation to online learning led students to face increased obstacles, as they had to focus on learning both new material and a new platform in an environment with more stress and distractions. For many students, it is easier to focus on college campuses, where there are libraries and quiet study spaces readily available.

Students were unaware of standard protocol for emergency situations such as that of the COVID-19 pandemic. Students are dealing with issues such as lack of motivation to engage in online schooling, attending school in different time zones, and issues with Internet connection [10].

“With online learning, professors have increased the workload given to students on average. It’s almost like they think now that we somehow have more time in the day, which may be true kind of, but I don’t appreciate it. It’s really overburdening to be constantly doing work to prove that you’re actually learning instead of taking the time to engage with material, which usually happens in a classroom.” –Aanya

There was a consensus among study participants that professors had increased the workload in the online setting, a factor that has led to an increase in burnout in academia [11]. Having to do the work for classes while also having to ‘prove that you're actually learning’ is frustrating, and students bear the brunt of the transition online. Furthermore, performing in academia during the pandemic meant sacrificing health and spending an unhealthy amount of time staring at a computer screen.

“Sometimes, I feel like my academic experience is just fit within my 13-inch screen, which is not what I had planned. I definitely feel in some ways I’ve lost the collaborative experience of learning.” –Rani

Upperclassmen expressed frustration toward their college experiences being cut short. Students felt the loss of senior year experiences, many wanting an in-person academic year to finish their baccalaureate education. The virtual environment made it more challenging to concentrate in school and to secure letters of recommendation necessary for many seniors interested in attending graduate school. A senior who hopes to go to law school shares her experiences of navigating virtual schooling while also trying to be present for her family.

“School is totally on the back of my mind because I’ve moved back home, and graduation has gotten postponed. I was supposed to be working on my senior’s honors thesis. I literally have no motivation to do that at this point because 1)the culmination of doing an honors thesis is being able to present it, and I probably won’t be able to present it. The work I’m doing now is not research-based anyways, so that’s a whole other thing. With academia, I need to make sure that I’m here for my family, I’m making sure they’re safe, and also dealing with my own family problems at home. It’s really hard for me to focus at home. There’s just a lot going on. Not only geographically is it hard for me because I’m not in [my university’s campus]. I’m not in that environment of like, okay I’m at school. Also, the whole Zoom situation isn’t helping me at all. I learn way better face-to-face. Office hours aren’t the same. I just haven’t been connecting with professors. It’s just harder. I don’t like emailing as much as I like going face-to-face in office hours. I’m honestly concerned about letters of rec, for example. I don’t like this whole zoom office hours situation. One of the professors I have this semester, I want to get her letter of rec. It’s really hard because I have to talk to her through Zoom. I don’t know that, that’s kinda awkward for me. I don’t like that. I’m glad the whole pass/no pass situation is happening, but also there’s so many intricacies with that, especially for folks who are going to apply to grad schools, law schools, med schools. It just affects … it’s a really tough decision to make. Some folks want to boost their GPA, want to get letter grades for something.” –Maaya

The lack of motivation for students was felt widely, with as many as 76% of all undergraduates identifying a lack of motivation as their most significant obstacle in a survey conducted with over 20,000 undergraduates [12]. The timing of the pandemic is especially unfortunate for college seniors who were at such an integral part of their college careers when the pandemic hit, especially in instances when students who have worked toward being able to present a honors thesis and/or research projects had that opportunity taken away from them due to the stay-at-home orders.

In the virtual setting, students struggled to form relationships with their professors, and interactions felt more forced. There is a correlation between student-teacher relationships and students' academic achievement [13]. The opportunity to know students on a personal level becomes much more difficult when ‘office hours aren't the same’ through Zoom.

For many college students who were members of the class of 2020 and 2021, in-person commencements were canceled or postponed. Commencement ceremonies have traditionally allowed students to celebrate receiving their diplomas among classmates, friends, and family. One college counselor who works with many college students from South Asian backgrounds in the Bay Area attests that for many students in the class of 2020, not having in-person college commencements at the time of completion has affected students’ experiences in academia.

“I’ve spoken to students at UC Berkeley, UC Davis, UC Santa Cruz, San Jose State, and Cal State East Bay. It’s basically unanimous. Because that graduating class can’t graduate, it’s the most depressing, the most debilitating, and the most depleting experience they’ve had thus far in their lives. You work so hard at this prestigious university, and it’s not like you’re not going to graduate, obviously you’re going to graduate. It’s the idea that was the closure we needed to move onto the next stage in our lives. It doesn’t feel like it closed. It just feels like it segwayed into this today is tomorrow, tomorrow is the next day, you can’t even tell. I think all aspects of life have really come to a weird, awkward halt.” –Raahim

Furthermore, graduating seniors faced challenges post-graduation finding employment during an economic crisis. The pandemic has driven youth unemployment, mainly affecting people of color [14]. This inability to find work upon graduation will likely affect graduates’ long-term career prospects [15]. Many college graduates found they had to move back home. For the first time since the Great Depression, the majority of young adults lived with their parents [16].

Many students beginning college during the pandemic would do so remotely from their homes rather than from campus, making it harder to form connections with their peers [17]. Events such as Orientation Week or popular campus events occurred online, where students missed out on the traditional college experience. Lower class students saw how a large part of their college experience would now happen online, and there was a large amount of uncertainty surrounding knowing when college classes would resume in person. One student, a freshman when COVID-19 initially hit and an intended Political Science major, explains how her perspective on college shifted.

“Since this is my second semester of college, I feel like it’s really changed my interpretation of what the college experience is like since I’ve barely been here. Now, I see the college experience as one that is going to heavily use tools like Zoom and more webcast lectures as professors get more comfortable with it. I’m coming to appreciate [my university] for their more adaptable policies. I feel like a lot of my professors have been really good with extending deadlines or changing formats to help students. I know not everyone has had that experience. I see a lot more of the humane side of [my university’s] staff.” –Noor

College is a time many students look forward to. For students who start their college experiences virtually, they miss out on a lot of the experiences and time for identity exploration that undergraduate students are normally afforded.

South Asian students fall on a wide range of financial stability. While there are South Asian students who felt they had financial security despite the pandemic, many South Asian families struggled to weather the hits to their small businesses or navigating losing their jobs during this time.

“The South Asian community, when you look at them from an economic spectrum, they’re all over. On the flip side, we have a lot of low wage individuals as well. We have small business owners who are trying to rebound. We’re so spread out on the economic spectrum that our experiences with the pandemic have been so different.” –Aadil

For students who came from lower ends of the economic spectrum, there was added financial stress on top of the academic stress posed from both the rigor of their classes and the transition of learning in-person to online.

College students were no longer able to attend the social events that they normally would be able to attend. When in quarantine, having a social life is one of the aspects students miss most [18]. The earlier years of college are often when students make friends that last throughout college, and students now lose out on that opportunity as it is much more difficult to bond and form deeper connections with peers online. Social events ranged from club events to religious events (such as the Hindu event Holi, also known as the Festival of Colors) to leisurely meetings to get to know classmates.

“One of the first things that I remember is that Holi got canceled. I remember that was a big deal. My cousin who’s actually my roommate right now, she’s a year below me. She was supposed to have her first Holi experience [at my university]. She didn’t get to do that because it got canceled.” –Rani

Many experiences were canceled for students, including internships and study abroad programs. Students could no longer participate in many extracurricular activities (that were typically held in person), and their college experiences were limited. Social experiences/integration can be seen as critical for students' success. Among the main reasons for students’ success are the relationships they build and positive academic/social experiences on campus [19]. By losing out on many in-person college experiences, students lose the sense of belonging they would feel if they were at their college campuses. Furthermore, many students had to adapt to college plans different from what they had originally hoped, dealing with postponed or canceled study-abroad trips or internships.

There are also concerns about the quality of education received, given the online format. There are more distractions associated with online school, and the format is less regimented. There had already been concerns about whether US colleges are preparing students to be able to compete globally, when there is more of a concentration on boasting high graduation rates or hosting state-of-the-art centers, rather than focusing on what students are learning and the quality of their educations [20].

“The university needs to re-evaluate what it is trying to achieve if the same information that I’m learning on a Zoom call right now, I could have paid $50 on Coursera to learn for a whole semester, then there’s not really a lot of value in my degree. I don’t think that the university at an institutional level is thinking about that. They’re not thinking about learning as much as they’re thinking about grades, how to get people to not cheat, or whether we should have pass/no pass, or whether we should have default grading. They spend so much time on those nitty gritty details that they don’t think about what the learning process looks like or the assimilation looks like.” –Karamleen

Students noted an absence of the more collaborative aspects of their education. The pandemic has led to lost opportunities for networking. Furthermore, there has been an increase in student absences during the pandemic.

“I think the biggest impact would just be not being able to go into class and not having group labs or projects being in person. I think that just affects how much information you retain and the quality of collaborative work because it’s just harder to have meaningful conversation, ideation over zoom because not everyone’s participating. People don’t have their cameras or mics on. You just can’t engage people as much. I think some of my professors have a difficult time switching because there’s definitely still technical problems. I’d say it’s a slightly lower quality education, but at least it’s still there.” –Aakriti

Students feel they are missing out on aspects of their education that they had planned out and hoped for. The lack of engagement in Zoom classrooms has been widely felt by both students and teachers. Student engagement is crucial for success in college and can be used as a measure of academic achievement [21].

Many students reported dealing with “Zoom fatigue,” which is brought upon by attending multiple meetings or events online in a day and leads to exhaustion due to decreased mobility and increased cognitive load [22]. Interactions over Zoom can increase cortisol levels as participants can only see each other's faces and are not reacting in real-time, in comparison to in-person communications, where people in conversation can physically see each other and read body language, which, in contrast, can increase dopamine levels [23].

Out-of-state and international students faced unique challenges adapting to the pandemic. Out-of-state students faced the burden of moving to a different state and potentially taking classes in a different time zone than the one that they were located in physically. Throughout the pandemic, students felt more disconnected from their lives on campus. College campuses are communities of their own, and it is difficult for students to maintain their personal networks when they are attending college from a different state.

“It’s far more impersonal. I feel so removed from everybody, especially being out-of-state. I think, otherwise, people, acquaintances and friends I would have made in section or lecture are just not there. It’s very awkward because nobody knows the rules of engagement or anything over Zoom. Everyone is in their own sphere, looking at the lecture with video off and muted. It is hard.” –Abdul

The decrease in student participation with students' ‘video off and muted’ was common throughout university experiences. Some professors have never been able to see students and were teaching, without even having physically seen their students once. Measures sought out by professors to facilitate student discussion, such as creating break-out rooms, were generally considered ineffective. Students had to shift toward learning independently and longed for the college community that had once been more accessible.

International students were initially told that they must leave the US if colleges were to go fully online in fall 2020 [24]. Logistics of the next steps to remain as healthy and safe as possible during the pandemic were increasingly difficult, given the conflicting information international students were given and having to work out plans on a global scale. For many international students, the US education system is foreign, and college is entirely different from high school. This transition is much more difficult to make when not in person. Two international students who participated in this study (both originally from India), Karamleen and Aanya, shared their experiences in California higher education during the pandemic, with the added stresses of not knowing whether they would be allowed to stay in the US and being very far from their families during this difficult time.

“For international students, there has been a lot of panic since mid-February. Everyone’s confused about whether they should dip–if it’s safe for them to go home, if it’s safer for them to stay here. With all the things shutting down very abruptly, it left international students hanging because we couldn’t just get up and go home.” –Karamleen

Many international students had to weather the pandemic far away from their families. However, international students residing in the US also had to navigate the changing policies of whether they would be allowed to stay in the country, which was incredibly stressful to do on top of managing their academics.

“I think a lot of us had troubles getting back home. I hadn’t visited home in a year because of this pandemic. I had to go this time just because we had some problems back home. I wasn’t able to travel. There were a lot of restrictions on what you can and can’t do if you leave the country. There were a lot of visa problems.” –Aanya

When the initial policy mandating international students must return to their home countries was announced, there were many efforts made to ensure international students would be able to remain in the US. The policy was overturned; however, the confusion and unease international students felt impacted their academics, causing stress and anxiety. International students add to the diversity of US classrooms, bringing unique perspectives and skills to US soil. Ensuring international students' success in the classroom should be a priority, and efforts to make international students’ transition to online schooling as easy as possible in future disasters is necessary to maintain the quality environment of US education.

Students noted a lack of preparedness or planning in emergency situations on behalf of universities’ administrations. A pandemic will likely happen approximately three times a century [25,26]. So, the question remains why were universities not more prepared for a transition to classes online?

“One of the biggest issues on campus, we were reactive to these issues instead of proactive. We had golden opportunities to be proactive about this, in my opinion. I think about a lot of our emergency preparedness, after power outages, after the fires, we can be a lot more instructionally resilient to what’s going on, prepare our students, and have our professors be prepared for this. That’s not been something that’s been received well. It’s been really tough. That’s something I’ve been really worried about.” –Chand

Authorities need help taking pandemic preparedness seriously because situations such as these are only *potential* threats at the time of allocating resources [27]. In an event such as the pandemic, where classes must shift online, universities should have a plan in place prior and be proactive as opposed to reactive. Had students and professors known that the protocol would be to switch to Zoom classes beforehand in the event of a pandemic or similar situation, the transition that instructors and students faced across California higher education would have been much smoother. This situation extends beyond the pandemic, to situations like earthquakes or fires. Furthermore, there should be more funding allocated toward dealing with similar disasters, so institutions are more able to handle the predicaments associated with navigating running a university online.

Guidance on behavior that was appropriate on Zoom and how to make the most out of the virtual setting would have helped students acclimate better. Communication with students about how to navigate online learning, including testing, would have eased the transition between in-person and online learning. Effective communication for students would include concision, transparency, sensitivity, and the opportunity to ask staff/administration further questions [28]. A senior computer science major shares her experience in one of her classes.

“Right before we were going to have one of my CS midterms, the university put out a statement that they were not allowing proctoring, via any interface, so no Zoom proctoring for example, which is what my class was planning on doing. That ended up turning into a non-proctored, timed exam that the professor made more challenging because he wanted to curb the effects of cheating. I think the transition there could have been better in terms of communicating with students and instructors about how to take exams. I think that this is kinda an unprecedented situation, so it’s kinda difficult to see what to do. I don’t really blame the administration for that.” –Saanvi

Having changing plans on how students are to take exams in already less-than-ideal circumstances was confusing for students to navigate. However, students understand how the situation could be challenging from both ends.

“It’s a huge transition from writing on-paper tests to online tests, especially when you’re writing science papers. You cannot draw a hexagon with Paint. It’s hard. I hate organic chemistry so much because my professor didn’t take into account that I didn’t have the technology like an iPad to draw on my screen. I needed to print it out, which means I had to run to my nearest shop, print it out there, and bring my test back last semester. That was absolute hell, but I got through it. I think professors should consider things like that.” –-Aanya

Having guidance toward the transition between taking on-paper tests to online tests would have helped students. Furthermore, not all students have the same access to technology, making the test-taking experience more difficult for some students than others. This reflects a broader trend where students who lack Internet or devices have suffered major obstacles toward higher education [29]. In classes where some students may not have the same technology as others, professors should seek to accommodate, whether that mean allowing a few extra minutes for a student to regroup or the university allowing the student to rent out a tablet or iPad for the semester.

Throughout the pandemic, the uncertainty of when COVID-19 would end loomed over students, as they adjusted their expectations for what their college experience would entail.

“There’s definitely anxiety and stress related with your grades, academics, and how well you're going to do, since you don’t really know where/how long this is going to last. You lose motivation sometimes, you’re too worried about other things, which makes sense.” –Hajna

Continuing to go through the motions of obtaining a degree without knowing when and how the pandemic would be less intrusive in students’ lives was stress- and anxiety-inducing. There was much more uncertainty surrounding their schooling and everyday life.

Additionally, navigating the varying policies for different classes has been confusing for students. Many students, who have taken different courses of study, such as double majoring or taking on a major and a minor, must keep track of the different policies of the colleges and departments they are enrolled in, compounding students’ stress.

“Different professors even have different policies, it’s really weird adjusting to how every professor is approaching the situation. There’s not really a standardized way of approaching it, even though university-wide, they have made decisions. Every college is dealing with it differently. Every department is dealing with it differently. Each professor from a different class is dealing with it differently. It’s hard to understand, okay is this allowed? Is this not allowed?” –Maaya

As a result of the pandemic, learning for STEM students in fields such as engineering was drastically different. STEM courses are often where applications to course material are very hands-on. For basic science classes, often students have to watch the experiments performed by a lab instructor rather than doing the labs themselves. Many students, who need experience working in research labs, could not secure those experiences during the pandemic. Experience in research labs is often seen as critical for graduate admission in STEM programs.

“I think the transition online has not been extremely well thought-out. It could be so much more smooth for students that need a whiteboard to see how a graph is drawn or students who need help with math. You can’t really just show by slides. A professor can’t really teach by talking to us. That’s been hard for me. I don’t think I understood anything in my Environmental Econ class from an online lecture. It’s literally just a professor sitting in his living room with a small whiteboard trying to explain it. It doesn’t work. It’s super stressful. The content is hard already and now that we don’t really have the right way to learn it, it definitely gets worse.” –Karamleen

For students like Karamleen, it would have been more beneficial if STEM professors had been well-versed in using a tablet, so students could see graphs or figures rather than professors switching the method of learning from using a white board to slides. It is possible for schools to train their teachers to be able to use a tablet, iPad, or similar device to illustrate diagrams and connect to Zoom during lecture times. There was also a frustration amongst students regarding inflexible policies, such as not recording lectures for students or not shifting times for students who are taking classes from different time zones.

Within STEM education, student engagement declined, and stress levels increased [30]. Hands-on experience and experiential learning are pivotal to learning in the lab setting. Students enrolled in STEM undergraduate programs need to be able to leave college with skill sets that match the level of degree they hold. While some schools have evolved their curriculum to send students lab kits to still complete labs, there were still concerns shared by both students and educators regarding the rigor of education received [31]. A bioengineering major shares her insights on labs and discussions.

“I think discussion sections at this point can be held in person. It’s about 10–15 people, and we have a huge glade. For engineering and STEM students who need to see things to learn and do things to learn, I feel like labs and discussion sections should be made smaller and then in-person. Otherwise, these kids will literally break down buildings when they become engineers. We don’t want that.” –Aanya

STEM students had to rely on virtual learning to convey the same knowledge of the intensive in-person lessons prior to the pandemic. Through the transition online, there are concerns about whether students are learning actively and experientially. Furthermore, for disciplines such as healthcare and engineering, the work students typically do in lab-based settings is crucial for their future careers, as Aanya notes in regard to engineering. The pandemic has increasingly left students of color more vulnerable. This was especially apparent within STEM, with fewer people of color following through with finishing their STEM courses of education [32].

Over a year after the COVID-19 pandemic hit, many universities began to transition back to offering in-person classes. This posed another difficult hurdle for students to overcome as they obtained their baccalaureate educations. Many students struggled to make plans as decisions regarding whether instruction would be in-person, online, or hybrid were delayed as university officials took time to deliberate on the best actions moving forward.

“The adjustment back to in-person classes was almost as, if not equally difficult as the initial transition to online classes when COVID started. Professors and administration attempted to make courses and events flexible with a hybrid learning approach, but this either discouraged students from attending classes, or made it more difficult for students who had already prepared for fully online classes to adjust. This time period also witnessed a heavy strain on basic needs like housing for students — for instance, I started looking for housing much later during the year because the guidelines for a return to in-person instruction weren’t made clear earlier. I don’t think that students adhered as strictly to the guidelines—lecture halls and event spaces were still very crowded despite the ongoing pandemic due to the lack of socializing that new and current students missed out on during COVID.” –Mahnoor

While the transition back to in-person classes offered students more of the in-person learning experience they had hoped and paid for, it also brought added stress and fear of obtaining COVID that learning in the virtual environment offered protection for many students from.

There were also housing issues for students, many having to scramble to find a place to live after the university's in-person plans were announced. Upwards of 3 million Californians filed for unemployment during just the first month of the pandemic alone, leading to increased food and housing insecurity [33].

Knowing that COVID-19 guidelines were not followed across all students posed additional stress as students are aware that they may bear the brunt of other students’ actions if their peers are not following social distancing guidelines and catch COVID themselves.

“I think that the most stressful thing about the readjustment to in-person classes was the contingency procedures if [someone] did get COVID … There were definitely people whose grades had to be calculated differently or who were penalized for COVID related absences or delays.” –Abdul

When classes transitioned back in person, it was more difficult for many students to stay safe from contracting COVID. There were instances where contracting COVID penalized their performance and grades, which was an unfortunate reality of attending university in person during the pandemic.

“[It was stressful] keeping up with a “live” pacing in the classroom. With lecture recordings normalized, the inability to pause/rewind made it so I’d have to keep up with/ relearn how to keep up with the pace of a classroom.” –Aadil

When students began to adjust to attending classes in person again, this came with the added stress of no longer being able to watch lectures at their own pace or having past lecture recordings to reference while studying for exams.

In addition to the pressures associated with college-level coursework and attending university during the pandemic, South Asian students faced external societal pressures to excel academically. Falling under the “model minority,” South Asian students feel the need to be compliant, to pursue competitive career paths, to become highly educated, or even to be good at math, all despite anti-Asian discrimination. Often, the external pressures South Asian students feel are internalized, and as students, they push themselves to receive the highest grades. While the pandemic affected students’ capabilities to learn, South Asian students continued to bear the burden of high expectations and excessive pressures of society, family, and educational institutions at the expense of their own well-being.

### 2.3 Mental health

As a result of the circumstances surrounding the pandemic, there has been an overall downturn in students’ mental health in South Asian communities. Students struggled to perform in the academic setting at the same capacity. Students experienced increased stress levels, with concerns ranging from the extent of the virus outbreak, exposure to the virus, and risk of contracting the virus for themselves and their families [34]. South Asians are at a higher risk than the general population if they contract COVID-19, as they are four times more likely than the general population to have heart disease or diabetes [35].

There are concerns about whether college counseling centers are prepared to deal with the dip in mental health college students are facing [36]. For South Asian students, there were further stresses, such as worrying about family overseas or explaining to elders in their native languages important COVID-19 related news.

Many South Asian countries were at a higher risk due to a larger population density, with people living in close quarters with one another. Risks were further exacerbated by weaker healthcare facilities, poor sanitation, higher amounts of poverty, and inadequate resources to follow social distancing guidelines [37].

“I was super worried for my family back in Karachi, Pakistan. The way coronavirus has affected those societies is terrifying. A lot of people are daily wage laborers, so quarantine was almost never an option. A lot of families live in one room. Families of 10 are going to be in one room. The only way they manage that, is that the older people go off to work, because you can’t really quarantine in that kind of poverty.” –Dafiya

The pandemic has affected the stability of many occupations. For daily wage laborers, such as those who drove rickshaws or construction workers, the pandemic meant having to go to work despite the stay-at-home orders and, in many cases, the loss of a job as travel and construction came to near halts. For students, this increased worry because the resources to combat the virus were not the same in their countries back home, and they could only provide a limited amount of help overseas.

For many students, the pandemic forced them to change their plans, leading to instability in their lives as students scrambled to find housing and jobs.

“I’ve definitely seen in my [South Asian] communities and in my close groups, there has been a decline in mental health. Most people are trying to figure out where to live. Some students don’t have the best situations at home. A lot of students aren’t able to meet their deadlines. Most students are struggling to find food. A lot of people have lost their jobs and reduced hours. A lot of issues surrounding mental health, financial stability, and education. A lot of people are terrified that their parents could have it and are facing mental complications.” –Sabiha

One of the barriers to South Asian students' accessing mental health resources is the stigma surrounding mental health within their own families and communities. South Asian students are less likely to seek help for mental health related concerns because of cultural pressures and stigma associated with mental illness [38]. Having or receiving mental health care or resources is considered a taboo topic. Prevailing attitudes toward mental illness in South Asian communities include believing mental illness to be God's punishment for a person's past sins, mental illness as untreatable, and children being unable to get mental disorders [39]. For many South Asian students who had to return to their homes, the COVID-19 pandemic only makes it more difficult for them to access mental health resources/support as the stigma surrounding mental health was more present. For many of these students, college was a way to seek out mental health resources away from home. Returning home has made it more challenging to seek out wellness resources despite a growing range of mental health issues.

Many South Asian students are first- or second-generation Americans and face the pressure of living up to often high expectations of immigrant parents. Knowing that parents have sacrificed to the extent of leaving their loved ones and families for opportunities offered in America can weigh heavily on South Asian students. Pressures South Asian students face can include parents living vicariously through their children or viewing their children's success as means to advance family pride [40]. Children who do not perform academically can be seen as bringing shame to families and feel guilt for failing to meet expectations [41]. The situation surrounding COVID-19 has exacerbated the stress many South Asian students already feel to perform strongly within academia.

“There’s a different type of social pressure associated with brown people, in general I guess: you have to be really academically capable, but then you really can’t be with COVID-19 because of all these other taboo things, you can’t say you have anxiety. You can’t say you have depression during this time. There’s that problem. Another problem is that it’s hard to take care of your mental health on top of that, especially if you’re coming from minority groups or from South Asian communities. You’re not allowed to go out or things that are not seen as normal I guess. It’s kinda weird … you can’t facetime your friends 24/7, after a specific hour.” –Hajna

The first step toward receiving mental health treatment is acknowledging a mental illness. When South Asian young adults internalize mental health issues such as anxiety or depression as ‘taboo’ as a result of their environment, then the barriers toward accessing and receiving treatment are compounded. In a research study conducted comparing Caucasian students and South Asian students enrolled in California higher education (with a sample size of 128), South Asian students experienced both higher levels of personal stigma toward persons with mental illness and an attitude toward receiving treatment that was less positive than their Caucasian counterparts [42]. In order to combat the higher levels of stigma associated with mental health treatment in South Asian communities, speaking of topics of mental illness must be normalized, and seeking out treatment must be seen as a healthy way for individuals to be the best versions of themselves.

College students had to face the stress of moving out of campus residence within a short time frame, with some universities giving students only days to move all their belongings [43]. College campuses were at high risk for the spread of COVID-19, and students bore the brunt of having to move out quickly.

“The residence halls have been primarily home for me. Given the proximity people have, people have to be displaced pretty heavily. They don’t have a place to call home at this point. There’s been displacement and financial struggles, accompanied with struggles back home.” –Daanika

Through the transition of classes back in person (under COVID-19 guidelines), the residence halls were high risk for students contracting COVID-19.

The stress of moving out was worse for out-of-state and international students, who did not necessarily have a second home close. Abdul, an out-of-state student, shares his experience.

“I’m still processing [the pandemic], I think. Everything’s been happening so fast. It’s really been a necessity to not think about it. I’m definitely not prepared. It was very abrupt. My move out from [my university] was a total mess. There was just a lot of fear and unknown stuff going on.” –Abdul

Thinking about other aspects of their lives was a common coping mechanism for students who partook in the study.

For international students, it was difficult to travel home because there were many airport closures. Dafiya, an international student, explains what it was like for her when schooling initially went online.

“Over here, I don’t have much of a South Asian community. My flat mates are all Californian, so they headed home which means I was all alone when the quarantine started. It was also kinda lonely.” –Dafiya

Students have struggled heavily with isolation during the pandemic with the virtual format of schooling. Human beings are social, and social networks allow people to thrive in society [44]. There is a vast body of research linking social isolation to having significant impacts on mental and physical health [45]. There is even evidence to suggest that social isolation is linked to an increased risk of premature death, similar to the risks associated with smoking, obesity, or being physically inactive [46].

“A lot of my friends have problems with mental health, not being able to handle the isolation and time away from people that well. Not being able to balance the stress and pressure of academic and work life, with now not having personal time and space. Especially for people who are staying at home and with their families, having more family problems and health concerns. There are very few places to get help.” –Aakriti

For many South Asian students, college offered a safe haven to seek out mental health resources, as mental health issues are generally stigmatized in South Asian communities. The transition to adjusting to college without the same resources and social circle was tough.

“I’m friends with lots of Muslims Alhamdulillah. I think that one thing that affects a lot of people is not being able to connect on faith-based issues because you can’t see each other anymore. That’s definitely a big thing. A lot of people are worried about Ramadan and how it’s going to be like. They're worried that it will be harder than usual. They won’t be able to see their friends, do the kind of things that typically were what they looked forward to in the month.” –Sabburah

For students who celebrated religious holidays, there was an understanding that to protect their own and their community's health, social distancing guidelines must take priority. For Muslims, the community-based iftars and in-person Eid prayers had to be adjusted to abide by public health guidelines.

Among the South Asian community, there is a push toward holistic approaches to deal with illness. While these methods do work for some conditions, there are no holistic approaches that have been proven to be effective against COVID-19. It is imperative that populations be educated on how to best combat the spread of infections in situations such as the COVID-19 pandemic.

There was a substantial consensus among study participants that mental health issues need to be addressed more commonly across South Asian communities, both more generally and especially now with the added stresses and pressures during the pandemic.

“We don’t have very robust conversations about mental health within our communities. We never have. We, as a generation, are pushing our parents and our uncles and our aunts toward having those conversations because not only do they have these horrific intergenerational traumas they’ve never faced, but we also have our own mounting series of mental health issues that they fail to understand. I think that’s interesting because so many of us have been forced to come home, live with our parents, and then deal with isolation. While some of us have very supportive and welcoming family structures, others of us are dealing with a series of mental health issues, on top of feeling isolated, on top of being in a South Asian household that doesn’t understand that dynamic.” –Aadil

Furthermore, many South Asian students feel the onus of educating family members to help them understand the ongoing situation of the pandemic and how that alters their daily life. This student notes, as a Sikh South Asian student, how difficult it was at the beginning of the pandemic to confront older members of their family, explaining why they could not attend Gurdwara, a place of worship.

“A lot of our elders go to the Gurdwara, our holy place/place of worship. Hey, we can’t go there. Gurdwaras have to close because it’s a public health issue. I think a lot of folks aren’t understanding and are still going to each other’s houses and stuff.” –Maaya

In educating South Asian communities, there is an uphill battle and a need for information to be given in individuals’ native languages. Furthermore, there needs to be a consensus among information to ensure individuals take stay-at-home orders seriously.

Due to the language barriers faced by many South Asian elders, it is challenging to effectively communicate and educate on the restrictions regarding the pandemic. South Asian students, well-versed in both English and their native languages, bridge the gap between news media and their family members.

“[A lot of my family] only speak Punjabi, so a lot of the language resources that they have, we don’t have as many language resources, at least from what I’ve seen in California. It’s been really hard to get them to understand the gravity of the situation.” –Maaya

The added stresses college students face during the pandemic have had a devastating effect on their mental and physical health, with some students even resorting to suicide [47,48].

“COVID-19 has also affected my mental health and my peers' mental health immensely. Our academic performances have been deterred by the fact that we do not get to socialize and are confined into the spaces of our home. It’s difficult to focus when there is a pandemic.” –Taz

South Asian students must juggle these responsibilities in addition to the rigorous academic requirements set out by higher education. Many participants expressed frustrations brought on by the pandemic, including discontentment having to navigate school and extracurricular activities through Zoom, having had to alter to a route of education different than the one they had signed up for, and difficulties in forming relationships with peers and instructional staff.

Furthermore, South Asians more commonly have pre-existing health conditions, such as hypertension, obesity, or heart disease, and are at greater risk of being adversely impacted by COVID-19 [49]. It is essential for South Asians to take extra precautions knowing they are at greater risk.

“At first it was a bit scary for my family and I as we didn’t know what was going on and how long we would have to be in lockdown. Now we have all bought masks and hand sanitizer and make sure we all carry our masks with us at all times and even wear disposable gloves when we go outside to grocery stores. When I get back to my car, I make sure to put on hand sanitizer. Also, when I get back home, I make sure to change my clothes and shower. It may seem extreme, but it’s better to be cautious especially with a deadly virus going around. There is so much uncertainty going around about when a vaccine will be released or when my school will open again, and it is getting stressful.” –Yalina

Once aware of the issues and potential risks, South Asian students took on the personal responsibility to take safety measures for themselves and for the betterment of public health. For many who had sacrificed a lot to be able to pursue a college degree, not having the in-person college experience and not knowing when they would be able to go back to college was taxing.

“Being a Bangladeshi-American, I have seen people from my community suffer during this COVID-19 pandemic from not being able to pay rent or provide for their families to being really sick. This pandemic has really brought out the economic inequities that we, as a society, have been facing for decades now. While wealthy people are able to get their hands on a test, there are families and my close family friends who aren’t able to get a test for COVID-19.” –Taz

The pandemic highlighted inequities between higher and lower classes, where wealthy people found themselves in the position to be more ‘able to get their hands on a test’ and, similarly, had more access to better trained doctors and the best treatments. This extended to schooling, where parents with lower education levels felt less confident helping their children with school [50]. At the university level, students from lower income backgrounds struggled to focus on school with additional stresses of lack of technology, food insecurity, and housing instability [51].

In India, the B.167.2 variant ravaged communities, leading to a surge of COVID cases and deaths. There were reports of whole villages becoming infected [52]. Many South Asian students were concerned, knowing the health systems overseas did not have the same capacity to deal with the exacerbated situation surrounding the pandemic.

Students expressed disappointment with how governments in South Asia handled the COVID crisis. Furthermore, South Asian students experienced fear when they had not heard from their families back home. There is a feeling of helplessness when students have more access to remaining healthy here but whose hands are tied when it comes to how they can help their families overseas.

“The situation has definitely affected my daily life, and that of my family’s. Every day we see [COVID-related news] online about the dire situation in India, and we feel scared for our family members that are living there. I see that lots of the messages and calls back and forth to India are about COVID-19 and telling people there to be safe. Some of my close family in India even had a COVID-19 scare, which was stressful for us here since all we could do was call and wait for the test results. So I think that what’s happening in India is a constant presence in my daily *life.”* –Aadi

In April 2021, India's infection rates set records, with upwards of 300,000 positive tests each day of the week [53].

Losing family members to COVID-19 overseas posed immense stress to South Asian students. In many South Asian countries, there was not enough room for burials to take place. In Sri Lanka, Muslims were cremated even though this is against Islamic practices [54].

“I think most of the support for the South Asian community needs to be donations for medical supplies, vaccinations, and better health guidelines for people to be socially distancing and wearing masks properly. I am kind of disappointed in how the Indian government handled the health and safety of the population after they relaxed social distancing and quarantine guidelines. Since they even encouraged people to travel for religious festivals and lots of places in India continued to have large gatherings under the presumption that the threat was over, it led to this situation right now. Institutionally, I think there needs to be a stronger focus on getting enough supplies and regulating movement of the population like they were doing before.” –Aadi

With the even more limited resources due to the critical situation in South Asia, there has been more blatant discrimination and fraud to hurt people in this already difficult time further. Students worry for their families back home, especially whether they will be able to access healthcare during the pandemic.

“The COVID-19 pandemic has affected my dad’s family—his sister passed away recently in Hyderabad while on oxygen. Even though she tested negative for COVID-19, she displayed all the symptoms. Thinking of her story reminds me of the intense discrimination that Muslims in India have faced: the healthcare system not only is inaccessible and hazardous for the most vulnerable, but it is also not a space that breeds trust amongst physicians and patients. I am also reminded of the severe lack of oxygen tanks and millions of families in India who do not have access to any care, and in a lot of ways I am grateful that my aunt had a proper burial, which many families can not afford there. This undoubtedly has been a difficult time for my father, and institutions at the very least can and should raise awareness about the ongoing cases not just in India, but in the wider South Asian subcontinent.” –Mahnoor

In India, many sought to profit off of the pandemic, with as much as a 500% increase in prices of thermometers and oximeters [55]. With the increasing religious tensions in the region, many South Asian students worried for their family and experienced loss that could have been avoided. In South Asia, there was a severe lack of equipment to be able to handle the health emergencies that they were tasked with.

“Our community didn’t experience damage even close to what other communities did. My home is India. It was horrible because our population density was really high. There was a complete lockdown for almost three months. It’s getting very dystopian. Those few months were horrible. A lot of social problems, especially for women’s independence. 70% of women owned businesses shut down.” –Aanya

Businesses led by women were hit disproportionately during COVID-19 [56]. Women are more likely than men to leave their work responsibilities to care for children and take on other domestic responsibilities [57]. Furthermore, there were more issues with women's health and independence with the increase in domestic violence during the pandemic, especially since domestic violence is an issue prevalent in South Asian communities [58,59].

With the transition back to in-person schooling, some students struggled to adjust to in-person interactions with others.

“Social anxiety, as it was odd, is back in shared co-learning space with others and certain collaborative behaviors have to be re-learned. There was a definite fear of contracting COVID, as this would bar you from participating in lecture/discussion and potentially cause one to fall behind in class.” –Aadil

There was a high prevalence of social anxiety throughout the pandemic [60]. After completing their courses through Zoom, students had to relearn interactions with others, with the added stresses of social distancing guidelines. Overall, South Asian students faced a multitude of mental health concerns exacerbated by the pandemic, and these issues are more difficult to overcome when mental health issues are stigmatized within the South Asian community.

### 2.4 Misinformation

Throughout the pandemic, it has been important to be aware of ongoing developments relating to the pandemic, of the correct guidelines to abide by in this ongoing public health crisis, and to not cause any unnecessary anxiety due to false information. The spread of misinformation is dangerous in situations related to healthcare, where misinformation can prevent effective care and even threaten the lives of individuals [61]. Misinformation during the pandemic has ranged from offering fake prescriptions or remedies for coronavirus to misinformation based on religious politics [62]. Social media has played a large role in the spread of misinformation [63]. Through social media, individuals can easily repost the information they see without necessarily verifying its accuracy. The misinformation surrounding the pandemic has been recognized as concerning, with the WHO referring to the phenomenon as an ‘infodemic’ [64].

“In general, there has been a lot of misinformation that has been spread about COVID-19. Especially on social media, people will repost the first thing that they see, what are the symptoms of COVID-19. I’ve seen so many posts: one post will say dry cough and a cold. The other post will say if you have pneumonia, you have COVID-19. There’s so much misinformation about it. I think that’s definitely important to address … maybe just centralizing all the information out there and fact-checking. My little brother and kids his age(10/11 years old) have access to the Internet and have access to a computer and are expected to adjust to the whole virtual class thing, especially if there’s the youth that are growing up in COVID-19. A lot of people say it might be called Generation C; it’s important that we spread the right information that it’s a pandemic” –Mahnoor

The consequences of misinformation are devastating. After a public figure suggested disinfectants could be helpful in combating coronavirus, there was an increase in incidents of accidental poisonings [65]. Impacts of misinformation were severe, with cases such as a preschool-aged child ingesting hand sanitizer to the extent of falling and vomiting and a woman consuming groceries coated with bleach [66].

Within South Asian communities, there has been misinformation used as fear-mongering. Many South Asians keep forwarding mass emails and texts without first checking the credibility of the source. This has, on many occasions, created widespread panic since the onset of the pandemic.

Further contributing to the issue of misinformation, on platforms such as YouTube, videos are often created as clickbait. Rather than educating the public, these videos disseminate falsehoods, causing confusion among those who have consumed such material. This has led to efforts toward creating computational tools for the automatic detection of misinformation [67].

“This brings back so many anecdotes with my grandmother when COVID first started. Those Facebook groups and WhatsApp, she takes everything for surface value and for what it is. I think it’s a bit of a generational thing as well. Some of the older generations, some of the more dense immigrant populations in our family have been victims of misinformation because of the fact they don’t have the ability to contextualize everything the way we do.” –Aadil

The population most vulnerable to believing misinformation is South Asian elders. The onus often falls onto South Asian students of college age to educate against misinformation while being mindful of the different experiences of South Asian elders, who may not be as privileged to be able to contextualize information. It is an important aspect of South Asian culture to respect elders, and so South Asian students must educate older generations against misinformation while also knowing their place within the family. This can be especially stressful for students in addition to their academic responsibilities.

“I feel like there’s a lot of misinformation that’s spreading on the Internet. You don’t really know what’s real and what’s not real. I’m still struggling to figure out what I should abide by and what I should discard as fake news.” –Mahati

The amount of misinformation present, especially during a global pandemic, can make it extremely difficult to ascertain when news is relevant. The need to put aside ‘fake news’ and have the necessary public health information was crucial for students to be able to focus on their studies while staying healthy.

“There’s a lot of misinformation everywhere. That’s just one problem our community has. A lot of us don’t speak the same language so it’s like a language barrier.” –Hajna

A lack of language access in healthcare systems can cause limited English proficient (LEP) patients to receive worse healthcare, and as many as 25 million individuals in the US are LEP [68]. Language barriers can further make it more difficult for consensus in public health measures. The language barrier is not just between South Asians and English-speaking Americans but also within South Asian communities, as different people speak different languages.

“Religion is very important in our community. I understand people are missing a lot of key social aspects in our religions. I miss that too. I understand, but I think some people have been cavalier about it … Oh, you know, if we go to prayer, we won’t get coronavirus. It’s like no, tie your camel first. It’s not an excuse to endanger yourself and other people, which I think some South Asians have been doing in my community.” –Abdul

While religion is traditionally a large part of South Asian culture, individuals must be educated on how to practice in a way that does not endanger individuals’ health. It would be helpful to work with religious leaders to come up with strategies for individuals to still be able to practice their faith while also being serious about public health safety guidelines and remaining cognizant of the consequences of large in-person gatherings.

Misinformation has also led some to doubt vaccine efficacy, which is incredibly dangerous and can lead to deaths that could have been avoided [69].

In order to mitigate some of the issues caused by misinformation, students supported the idea of a team of epidemiologists or doctors, well versed in South Asian languages, who would regularly talk to the community about medical advice and inform community members about the latest information. This need for multilingual doctors extends to health care, where language-concordant care has proven to improve the likelihood of patient health/safety and close the gaps of health inequity [70].

“We have an organization here in America to retain family ties and stuff like that. Our leadership has been doing a really good job of providing support for us through COVID. A couple weeks ago, a bunch of doctors in our org held a telecasted Q&A. Over 100 people tuned in. It really helped people calm down a little bit. My parents watched it. He emailed us the transcript after. It was a pretty good way of centralizing information for us and from people who we really trust in our community.” –Noor

Having trusted medical workers communicate pertinent information needed for safety to South Asian families would help combat the amount of misinformation spread. These interactions on a regular basis would help alleviate some of the stresses South Asian community members faced during the pandemic and would help prevent the spread of wrong and potentially harmful information.

“I think misinformation has been really bad, in India especially. There’s one Indian channel now that’s dedicated to literally 24/7 news. Every hour, it plays the same thing. I think the hope is if people go to that channel, people will stop doing clown things because they’ll see a doctor telling them what to do.” –Aakriti

To combat misinformation, trusted officials should communicate clearly and consistently to the public. For example, people generally trust doctors and the expertise that comes with training to receive their white coats.

“We live in a country where misinformation is so rampant. You can go to different resources and engage in confirmation bias. For me personally, the best sources are the nonpartisan ones, the sources that literally have no goal except communicating to you the facts in order to protect yourself. I resist the temptation to spread the information I find on social media, Twitter, Instagram, Facebook.” –Aadil

Interacting with nonpartisan news outlets is crucial as individuals tend to accept information that aligns with their own views [71]. This leaves individuals more vulnerable to acting on misinformation as well as spreading it to their social media feeds. With the continued growth of social media, it is crucial to limit the amount of misinformation, especially in times such as the COVID-19 pandemic.

### 2.5 Religious tensions

In much of South Asia, there is worsening religious tensions. Within Sri Lanka, there is a rise in extremist Buddhist nationalism, where Christian and Muslim minorities have been targeted [72]. In Pakistan, there has been a rise in religious-based hate crimes against Hindu, Christian, Shia Muslim, Sikh, and Ahmadi minorities, where individuals of these backgrounds were even denied food aid needed during the pandemic [73].

There is consensus among The United States Commission of International Religious Freedom that there are growing religious tensions in India that endanger the freedom of religion, with one commissioner listing India as a “country of particular concern” for its infringement on religious freedom [74]. Existing prejudice and religious discrimination have been amplified due to the pandemic, with an increase in violence toward minority communities [75]. In India, Muslims are being blamed for the spread of the virus, in one case, where Muslim men are targeted, beaten, made to apologize for spreading the virus [76]. One government hospital in India segregates Muslim patients with COVID-19 from Hindu patients [77]. In Uttar Pradesh in India, Christians were targeted by Hindu militants, where they were threatened if they continued to gather for prayer and several worshipers needed urgent medical attention as a result of physical abuse [78].

Within South Asia, there has been an increase in religious-based hate speech and the use of Internet shutdowns to block access of religious communities to health information [79]. In Rakhine and Chin states, the government of Myanmar has denied more than 1,000,000 residents access to the Internet [80]. During the COVID-19 pandemic, this is especially harmful, given how crucial staying informed of public health safety guidelines is to community health.

Students residing in the US are aware of religious animosity and speak to their family members’ experiences as well as their own experiences in the states.

“I think back home in India, I’ve seen people really afraid, given the political climate there with Muslims, people are really afraid because they think tests won’t be administered to that community because of the existing Islamophobia. I think on top of the public health crisis, people think that the additional layer of Islamophobia will further impact that community. People won’t get tests. People won’t get treated. There’s already healthcare discrimination against Muslims. I think people think it will add another layer on top of that. Here in the United States, I think people are a lot more chill because I think the problem here isn’t quite as bad as what’s going on in India. People here are worried about relatives back at home, but they’re not directly affected by what’s going on.” –Aaquil

South Asian students are aware of the religious tensions and worry about families residing there. There are concerns about international rights and the discrimination foreign governments put on minority citizens. Students recognize that they are privileged not to face discrimination to the same level as individuals outside of US borders.

Minorities also face discrimination and exclusion in regards to their citizenship, leaving them especially vulnerable, where minority citizens in South Asian countries are met with a denial of their basic human rights, a denial of healthcare services, or an inability to access information in their native languages [81].

“I was doing some research on this alone, and I looked in what India, in India specifically there was the Epidemic Disease Act, passed in 1897. It was imposed when Britain still colonized India. It’s still in place now. It’s very vague, but essentially it gives local authorities the power to do whatever they can to the communities in order to contain whatever disease that's going around. At the time that it was passed, it was in response to the bubonic plague, but now it’s in place specifically because of COVID-19. That in itself is already basically placing these authorities in a much higher power and establishing a superiority between the general public and the government, which in itself is already an issue. In December of last year, the Citizenship Amendment Act was passed, which basically fast tracks Indian citizenship for a lot of religious groups excluding Muslims. It’s the first time in India that there are these laws where religion is used as criteria. Both of these pieces of legislation passed in a similar time frame, it specifically places Indian Muslims in a lot of scrutiny and a lot of suppression.” –Mahnoor

The formation of laws to target religious minorities shows the far-reaching effects of religious discrimination. The Citizenship Amendment Act allows for individuals of Hindu, Sikh, Buddhist, and other religious majorities from the neighboring countries of Afghanistan, Bangladesh, and Pakistan to seek refuge in India and fast track their citizenship. This law stipulates that only Muslim refugees be considered illegal, specifically targeting Muslims and using religion as a criterion for citizenship [82]. Furthermore, the Indian government pushes for a nationwide citizenship verification process, creating a National Register of Citizens, leaving many Muslim communities distraught, fearing the loss of citizenship [83].

The discrimination reflects religious tensions on a global scale and extends to the US. According to a survey by the Institute for Social Policy and Understanding, among all religious groups, the highest percentage of respondents who had reported a lifetime suicide attempt religiously identified as Muslim [84].

“If we think about the immediate community here in the United States, one of my dad’s closest friends was laid off from his company. They want a more Hindu centric workforce in their office. And my dad’s friend is Muslim. That’s how it’s playing out here. Especially during this coronavirus pandemic, the last thing you want is your job being taken from you because there’s already so much financial/economic insecurity. It’s interesting to see the parallels, comparing countries, just how much it’s impacted us as a community.” –Mahnoor

Even within the US, participants have seen how not coming from an accepted religious majority can be harmful in terms of employment. Religious discrimination is far-reaching and can be a lot for South Asian students to deal with, in addition to the current financial uncertainty.

This participant notably observed that being a part of the South Asian community comes with some privilege, given the status as a “model minority.” However, coming from a non-Hindu background could be seen as more marginalized.

“I’ve been thinking a lot if I identify as a [person of color]. I’m not sure. In some USA communities, I am white adjacent, until they find out I’m from a Muslim background and not a Hindu background, which is when I can feel my privilege being lost. I’m still thinking about whether I can claim the POC label.” –Dafiya

In the US, there has been a rise in hate crimes against religious minorities such as Muslim Americans, Sikh Americans, and Jewish Americans [85]. For South Asian students, navigating the discrimination toward their communities adds significantly to social stress and has increased during the pandemic. South Asian students from minority backgrounds hoped for more just practices in the future despite differing beliefs and for members of the South Asian community from different religions to be able to coexist peacefully.

### 2.6 Other considerations

While the pandemic has had many negative consequences for students’ educations, there were some positives for students as well. Students noted having more flexibility in their daily schedules without the commute time walking between classes. With recorded lectures, there was more opportunity for students to pause and/or replay lectures, allowing students to engage with the course material at their own pace. Students were more easily able to attend office hours with the click of a Zoom link as opposed to walking across campus.

“For me, it’s been good to use my free time to focus on health and wellness. There’s a massive restructuring of what time even looks like now. I’ve been using YouTube workout videos to be active and mentally prepared. I also feel like the fact that I’m able to access information has been keeping me sane in terms of being in the know-how.” –Daanika

For many students, professors were understanding and accommodating during the spring 2020 semester, when classes initially went online. The pandemic also offered students a different perspective, with some participants noting a greater appreciation for their university experiences in person.

There was a consensus among the college students interviewed that platforms such as YouTube, TikTok, Netflix, etc. made the pandemic more bearable. A Pakistani American participant who is an out-of-state student shares how resources have helped him.

“Zoom and Facetime have been the staples of my life. Every day, my screentime gets higher and higher. It’s everything: classes, friends, anything and everything goes over to Zoom and Facetime. Online games, platforms, and educational tools are always good. News and entertainment, Netflix and YouTube, and having access to all these different platforms has been super, super important. I couldn’t even imagine going through something like this even 10 years ago, when these resources aren't available. It would have been completely different. There are apps, local news apps, that update me on the cases in the counties around me, how many people are contracting COVID generally. Those are always helpful.” –Abdul

Advances in technology today allow for students to more easily connect with one another, even if virtually.

Amongst Indian American participants in the study, particularly participants who came from a Hindu background, there was an acknowledgement of financial stability despite the economic turmoil.

“I come from an upper middle class suburb background, so a lot of the brunt of the coronavirus pandemic, I haven’t seen that fall on my immediate Indian American circle.” –Mahaati

For students who had economic stability throughout the pandemic, there is an acknowledgement of privilege. Students were able to contextualize the stresses of the pandemic, realizing that they had more resources to deal with disaster than some of their peers.

“Personally, I haven’t really seen my community impacted by the pandemic. Home life is going pretty well. [Our family] spends a lot of time together. We have a lot of dinner conversations together. I spend a few hours downstairs cooking for them. It has been pretty nonintrusive into our life.” –Gajpati

Students recognized the relationship between the pandemic and the overall health of their larger South Asian communities, but this was not as severe for their immediate friends and family.

## 3 Conclusions

The ongoing COVID-19 global pandemic continues to have negative consequences on students' educations. For South Asian students, many are worried about their families back home, the inability to physically be present for their families overseas, and feel helpless when the pandemic is beyond the control of governing authorities. This qualitative study found a relationship between the pandemic and the ability of South Asians to learn in the classroom, a decline in students’ mental health, anxiety related to misinformation, and difficulty managing religious tensions. The implications COVID-19 has had on South Asian students illustrate a need for institutions to be more accommodating toward students.

## 4 Future guidance & recommendations for policy and research

### 4.1 Addressing limitations of this study & offering guidance for future research

In order to overcome the limitations addressed in this paper in future research, some alternatives would be to have more researchers working on the project and to potentially acquire funding. These additional resources would allow for a more detailed study and quicker publication. The lack of previous research shows a literature gap and the need for more research to be conducted on South Asian populations. In order to avoid the social media bias, it is possible to acquire participants through passing out flyers to students on college campuses or having announcements sent out by college professors to students.

### 4.2 Potential future actions to take & recommendations

Educational institutions need to have contingency plans in the event of an emergency. The goal should be to minimize the impact on students’ educations and to create lesson plans that are more manageable given the added stress students are facing. Administrators must work with faculty and students to ensure that schoolwork remains manageable in uncertain times. Departments should coordinate to ensure that policies are uniform for students taking classes.

There needs to be more funding allocated toward higher education, especially for public universities, which are eligible for funding through the state and national governments. The UC System received a CARES Act allocation of $260 million, divided evenly between giving students emergency relief and covering costs the institutions have incurred. However, the $130 million that the UC system received to cover institutional costs is not enough to cover the $310 million spent by UCs [86].

International and out-of-state students must be a priority, given the many benefits of a diverse classroom. Educational systems should move toward the idea of a multicultural education, proposed by Banks [87]. Institutions should create culturally aware and inclusive spaces for students to be able to learn to their fullest capabilities. The curb-cut effect is a phenomenon that analyzes how when curbs were brought to the ground to help disabled individuals, it ended up helping mothers with strollers or travelers with luggage. The curb-cut effect illustrates how programs and incentives made to benefit vulnerable populations often end up helping all populations [88]. Similarly, initiatives proposed to help minority students will end up helping all students.

There needs to be an increase in relief packages for students who come from lower-income backgrounds. There also needs to be an expansion of healthcare options during the pandemic, as it is extremely important for individuals to know they have healthcare, in the event they or a family member gets COVID-19. For students who come from families of essential workers, there should be hazard pay calculated in their income potential for essential workers to reflect the risk of the jobs they continue to do.

The pandemic brought with it digital inequality, especially across undergraduate students who previously had access to campus WIFI and computers at university. For families who have a limited amount of devices for multiple family members to use/share, schools need to explore options to loan devices to students. For students who relied on university devices, schools should look to ensure that students have the same access they did prior to the beginning of the pandemic. For professors in STEM disciplines, it is beneficial to consider offering training to use tablets (or similar devices) to illustrate concepts for students to visualize during lectures.

In order to prioritize students’ mental health during this time, there needs to be an increase in preventative measures taken and proactive interventions, knowing the increased amount of stress students are currently experiencing. Crisis centers at colleges and universities need to be expanded, and there needs to be efforts to destigmatize mental illness in South Asian households. In order to combat social isolation, it would be helpful to ensure that universities are offering students virtual opportunities to connect. Furthermore, while expanding mental health facilities available to accommodate the many more students facing added mental health issues, it would be helpful for facilities to recruit more therapists or counselors specialized in helping individuals from minority backgrounds.

There needs to be mask mandates. Schools and businesses should only be allowed to carry at a capacity that is healthy for individuals. There needs to be increased access to testing, so individuals can identify quickly when they have contracted COVID-19 and quarantine, to protect the health of themselves, their family members, and fellow students. There needs to be an expansion of healthcare options, so students are not worried about both their families’ health and their own health beyond the risks already inherently present when living through the COVID-19 pandemic.

Within South Asian communities, there need to be efforts to combat misinformation. Social media companies can take on the responsibility of mitigating misinformation in relation to the pandemic. The World Health Organization took steps to address misinformation by designing infographics to help dispel some of the myths surrounding coronavirus [89]. There needs to be a concerted effort from community members and public officials to mitigate the spread of misinformation.

It is important for information and infographics to be readily available in languages understandable to South Asian elders, such as Hindi, Tamil, Bengali, or Sanskrit. Within healthcare, there should be accessible language interpretation, whether in-person or remote [90]. There needs to be a team of healthcare professionals, well versed in languages commonly spoken by South Asian individuals, who address the community and ensure that they are receiving the correct health information in such a critical time. Public officials should be briefed on their speeches and public messages to ensure all information received to the public is clear and could not be misconstrued to messages harmful to public health. Consistent, clear messaging is key.

Due to the pandemic, there is an increase in religious tensions. It is best for national leaders to preach tolerance of different religions and cultures. Religious minorities are the most vulnerable, and actions must be taken to protect individuals of these religions from prejudice and persecution. Lawmakers should ensure that policies consider minorities and their well-being/health, as these policies will end up benefiting all people, as demonstrated by the curb-cut effect.

## Author contribution statement

Zobia Quraishi: Conceived and designed the experiments; Performed the experiments; Analyzed and interpreted the data; Contributed reagents, materials, analysis tools or data; Wrote the paper.

## Data availability statement

Data included in article/supp. Material/referenced in article.

## Additional information

Supplementary content related to this article has been published online at [URL].

## Declaration of competing interest

The authors declare that they have no known competing financial interests or personal relationships that could have appeared to influence the work reported in this paper

## References

[1] Fauci A.S., Lane H.C., Redfield R.R. (2020). Covid-19 — navigating the uncharted. N. Engl. J. Med..

[2] (2023). We thought it was just a respiratory virus | UCSF magazine. https://magazine.ucsf.edu/we-thought-it-was-just-respiratory-virus.

[3] Nicola M. (Jun. 2020). The socio-economic implications of the coronavirus pandemic (COVID-19): a review. Int. J. Surg..

[4] Singhal T. (Apr. 2020). A review of coronavirus disease-2019 (COVID-19). Indian J. Pediatr..

[5] Magableh G.M. (2021). Supply chains and the COVID‐19 pandemic: a comprehensive framework. Eur. Manag. Rev..

[6] (2023). About the CARES Act and the Consolidated Appropriations Act.

[7] M. T. Nietzel, “Pandemic's Impact On Higher Education Grows Larger; Now Estimated to Exceed $120 Billion,” Forbes. https://www.forbes.com/sites/michaeltnietzel/2020/09/29/pandemics-impact-on-higher-education-grows-larger-now-estimated-to-exceed-120-billion/(accessed Mar. 16, 2023).

[8] L. Moscoviz and D. K. Evans, “Learning Loss and Student Dropouts during the COVID-19 Pandemic: A Review of the Evidence Two Years after Schools Shut Down”.

[9] D'Amato P. (2020). http://hechingerreport.org/coronavirus-accelerates-higher-educations-trend-toward-distance-learning/.

[10] UCLA Experiences During the Covid-19 Pandemic | UCLA Student Affairs Information & Research Office.” https://sairo.ucla.edu/by-topic/covid (accessed Mar. 16, 2023).

[11] Gewin V. (2021). Pandemic burnout is rampant in academia. Nature.

[12] NASFAA | Students Face Obstacles, Lack of Motivation in Transition to Remote Learning Amid Pandemic, Report Finds.” https://www.nasfaa.org/news-item/22637/Students_Face_Obstacles_Lack_of_Motivation%20_in_Transition_to_Remote_Learning_Amid_Pandemic_Report_Finds (accessed Mar. 16, 2023).

[13] Why strong teacher relationships lead to student engagement and a better school environment. http://Waterford.org.

[14] (2020). https://tcf.org/content/report/the-covid-19-recession-is-hitting-young-workers-especially-young-workers-of-color-the-hardest/.

[15] J. Tipograph, “Council Post: Why Your First Job Post-College Matters More Than You Know,” Forbes. https://www.forbes.com/sites/forbescoachescouncil/2019/05/24/why-your-first-job-post-college-matters-more-than-you-know/(accessed Mar. 16, 2023).

[16] R. Fry, J. S. Passel, and D. Cohn, “A majority of young adults in the U.S. live with their parents for the first time since the Great Depression,” Pew Research Center. https://www.pewresearch.org/fact-tank/2020/09/04/a-majority-of-young-adults-in-the-u-s-live-with-their-parents-for-the-first-time-since-the-great-depression/(accessed Mar. 16, 2023).

[17] T. C. S. J. Corps, “What's it like to be a college freshman during a pandemic? Students share their stories,” EdSource. https://edsource.org/2020/whats-it-like-to-be-a-college-freshman-during-a-pandemic-students-share-their-stories/645242 (accessed Mar. 17, 2023).

[18] Ezarik M. (2021). COVID-era college: are students satisfied?. High Educ..

[19] Department of Higher Education and Learning Technologies (2018). Texas A&M university @ commerce, United States and G. D. Caruth, “student engagement, retention, and motivation: assessing academic success in today's college students”. PER.

[20] Review: Charting Academic Drift: Academically Adrift: Limited Learning on College Campuses, by Richard Arum and Josipa Roksa by Mark Bauerlein | NAS.” https://www.nas.org/academic-questions/24/3/review_charting_academic_drift_academically_adrift_limited_learning_on_college_campuses_by_richard_arum_and_josipa_roksa (accessed Mar. 17, 2023).

[21] G. Inc (2019). https://www.gallup.com/education/267521/focus-student-engagement-better-academic-outcomes.aspx.

[22] University S. (2021). https://news.stanford.edu/2021/02/23/four-causes-zoom-fatigue-solutions/.

[23] T. Walker, “How ‘Zoom Fatigue’ Impacts Communication With Students | NEA.” https://www.nea.org/advocating-for-change/new-from-nea/how-zoom-fatigue-impacts-communication-students (accessed Mar. 16, 2023).

[24] Treisman R. (2020). ICE: foreign students must leave the U.S. If their colleges go online-only this fall. NPR.

[25] (2007). A severe pandemic is not overdue - it's not when but if | CIDRAP. https://www.cidrap.umn.edu/business-preparedness/severe-pandemic-not-overdue-its-not-when-if.

[26] G. Vince, “Global transformers: What if a pandemic strikes?” https://www.bbc.com/future/article/20130711-what-if-a-pandemic-strikes (accessed Mar. 17, 2023).

[27] Disaster response expert explains why the U.S. wasn't more prepared for the pandemic > News > USC Dornsife.” http://dornsife.usc.edu/news/stories/3182/why-u-s-wasnt-better-prepared-for-the-coronavirus (accessed Mar. 17, 2023).

[28] Communication during the Covid crisis: learning from our mistakes,” New America. http://newamerica.org/education-policy/edcentral/communication-during-the-covid-crisis-learning-from-our-mistakes/(accessed Mar. 18, 2023).

[29] CRPE (2020). https://crpe.org/the-digital-divide-among-students-during-covid-19-who-has-access-who-doesnt/.

[30] Wester E.R., Walsh L.L., Arango-Caro S., Callis-Duehl K.L. (2021). Student engagement declines in STEM undergraduates during COVID-19–driven remote learning. J. Microbiol. Biol. Educ..

[31] S. Johnson, “California science teachers look for new ways to bring hands-on experiments to students,” EdSource. https://edsource.org/2020/california-science-teachers-look-for-new-ways-to-bring-hands-on-experiments-to-students/633302 (accessed Mar. 17, 2023).

[32] Das L.T. (2020). OPINION: why school shutdowns are a disaster for science classes. http://hechingerreport.org/opinion-why-school-shutdowns-are-a-disaster-for-science-classes/.

[33] Reed S. (2022). Disparate impacts of COVID-19 disruptions for California college students. J. Stud. Financ. Aid.

[34] Wang F., Zhang L., Ding L., Wang L., Deng Y. (2022). Fear of COVID-19 among college students: a systematic review and meta-analysis. Front. Public Health.

[35] The Disparate Impact of COVID-19 Across South Asian American Communities – Asian American Policy Review,” Apr. 16, 2021. https://aapr.hkspublications.org/2021/04/16/the-disparate-impact-of-covid-19-across-south-asian-american-communities/(accessed Mar. 17, 2023).

[36] Anderson G. (2020). https://www.insidehighered.com/news/2020/09/11/students-great-need-mental-health-support-during-pandemic.

[37] Rasul G. (2021). Socio-economic implications of COVID-19 pandemic in South Asia: emerging risks and growing challenges. Front Sociol.

[38] Karasz A. (2019). Mental health and stress among South Asians. J. Immigr. Minority Health.

[39] Kishore J., Gupta A., Jiloha R.C., Bantman P. (2011). Myths, beliefs and perceptions about mental disorders and health-seeking behavior in Delhi, India. Indian J. Psychiatr..

[40] Bhattacharya G., Schoppelrey S.L. (2004). Preimmigration beliefs of life success, postimmigration experiences, and acculturative stress: South Asian immigrants in the United States. J. Immigr. Health.

[41] Mokkarala S., O'Brien E., Siegel J. (2015). The relationship between shame and perceived biological origins of mental illness among South Asian and white American young adults. Psychol. Health Med..

[42] Loya F., Reddy R., Hinshaw S.P. (2010). Mental illness stigma as a mediator of differences in Caucasian and South Asian college students' attitudes toward psychological counseling. J. Counsel. Psychol..

[43] Hess A.J. (2020). Harvard gives students 5 days to evacuate dorms over coronavirus fears—here’s what students have to say. CNBC.

[44] Why Being Social is Good for You.” https://www.southuniversity.edu/news-and-blogs/2018/05/why-being-social-is-good-for-you (accessed Mar. 17, 2023).

[45] The risks of social isolation,” https://www.apa.org. https://www.apa.org/monitor/2019/05/ce-corner-isolation (accessed Mar. 17, 2023).

[46] (2022). Loneliness and Social Isolation Linked to Serious Health Conditions.

[47] Chatterjee R. (2021). https://www.npr.org/sections/health-shots/2021/02/02/962060105/child-psychiatrists-warn-that-the-pandemic-may-be-driving-up-kids-suicide-risk.

[48] Manzar M.D., Albougami A., Usman N., Mamun M.A. (May 2021). Suicide among adolescents and youths during the COVID-19 pandemic lockdowns: a press media reports-based exploratory study. J. Child Adolesc. Psychiatr. Nurs..

[49] Chronic Conditions Among U.S. South Asians in the Context of COVID-19 - SAPHA.” https://www.sapha.org/resources/chronic-conditions-among-u-s-south-asians-in-the-context-of-covid-19/(accessed Mar. 17, 2023).

[50] Haelermans C. (2022). Sharp increase in inequality in education in times of the COVID-19-pandemic. PLoS One.

[51] Olfert M.D. (2021). Struggling with the basics: food and housing insecurity among college students across twenty-two colleges and universities. J. Am. Coll. Health.

[52] A. of S. A. Peoples (2021). https://www.imb.org/2021/05/05/praying-india-covid-19-crisis/.

[53] Thiagarajan K. (2021). Why is India having a covid-19 surge?. BMJ.

[54] (2021). Bangladesh must speak out against forced cremation of Muslims in Sri Lanka. Amnesty International.

[55] UP seeing massive scam of 500% increase in prices of thermometers, oximeters, claims AAP.” https://theprint.in/india/up-seeing-massive-scam-of-500-increase-in-prices-of-thermometers-oximeters-claims-aap/501531/(accessed Mar. 16, 2023).

[56] Torres J., Maduko F., Gaddis I., Iacovone L., Beegle K. (2023). The impact of the COVID-19 pandemic on women-led businesses. World Bank Res. Obs..

[57] Goldstein M., Gonzalez P., Papineni S., Wimpey J. (2022).

[58] Kourti A. (2023). Domestic violence during the COVID-19 pandemic: a systematic review. Trauma Violence Abuse.

[59] Bhandari S., Millner U.C. (2022).

[60] McLeish A.C., Walker K.L., Hart J.L. (2022). Changes in internalizing symptoms and anxiety sensitivity among college students during the COVID-19 pandemic. J. Psychopathol. Behav. Assess..

[61] Wang Y., McKee M., Torbica A., Stuckler D. (2019). Systematic literature review on the spread of health-related misinformation on social media. Soc. Sci. Med..

[62] Al-Zaman Md S. (2022). A thematic analysis of misinformation in India during the COVID-19 pandemic. Int. Inf. Libr. Rev..

[63] Schmidt A.L. (2017). Anatomy of news consumption on Facebook. Proc. Natl. Acad. Sci. U.S.A..

[64] Managing the COVID-19 infodemic: Promoting healthy behaviours and mitigating the harm from misinformation and disinformation.” https://www.who.int/news/item/23-09-2020-managing-the-covid-19-infodemic-promoting-healthy-behaviours-and-mitigating-the-harm-from-misinformation-and-disinformation (accessed Mar. 17, 2023).

[65] M. Perez, “Accidental Poisonings From Bleach And Disinfectants Continued To Rise In April,” Forbes. https://www.forbes.com/sites/mattperez/2020/05/12/accidental-poisonings-from-bleach-and-disinfectants-continued-to-rise-in-april/(accessed Mar. 16, 2023).

[66] Chang A. (2020). Cleaning and disinfectant chemical exposures and temporal associations with COVID-19 — national poison data system, United States, january 1, 2020–march 31, 2020. MMWR Morb. Mortal. Wkly. Rep..

[67] Hou R., Pérez-Rosas V., Loeb S., Mihalcea R. (2019). http://arxiv.org/abs/1909.01543.

[68] Molina R.L., Kasper J. (2019). The power of language-concordant care: a call to action for medical schools. BMC Med. Educ..

[69] Misinformation fueling vaccine hesitancy, PAHO Director says - PAHO/WHO | Pan American Health Organization.” https://www.paho.org/en/news/21-4-2021-misinformation-fueling-vaccine-hesitancy-paho-director-says (accessed Mar. 17, 2023).

[70] Molina R.L., Kasper J. (2019). The power of language-concordant care: a call to action for medical schools. BMC Med. Educ..

[71] Pereira A., Harris E., Van Bavel J.J. (2023). Identity concerns drive belief: the impact of partisan identity on the belief and dissemination of true and false news. Group Process. Intergr. Relat..

[72] Buddhism and State Power in Myanmar | Crisis Group.” https://www.crisisgroup.org/asia/south-east-asia/myanmar/290-buddhism-and-state-power-myanmar (accessed Mar. 17, 2023).

[73] COVID-19 (2020). https://jia.sipa.columbia.edu/online-articles/covid-19-catalyst-minority-exploitation-pakistan.

[74] H. Akins, “The Citizenship (Amendment) Act in India”.

[75] (2020). COVID-19 strikes South Asia's minorities at a time when their rights are already threatened across the region, as flagship annual report makes clear. Minority Rights Group.

[76] Sarkar S. (2020). Religious discrimination is hindering the covid-19 response. BMJ.

[77] P. MN, “‘Apartheid’: Hindu, Muslim wards in Gujarat hospital.” https://www.aljazeera.com/news/2020/4/16/india-hospital-segregates-muslim-and-hindu-coronavirus-patients (accessed Mar. 16, 2023).

[78] Sabatier L. (2019). India: growing intolerance and hate toward religious minorities. 21Wilberforce.

[79] K. Lavery, “The COVID-19 Infodemic and its Impact on Religious Communities in South Asia”, [Online]. Available: https://www.uscirf.gov/sites/default/files/COVID19%20Infodemic%20Impact%20in%20South%20Asia.pdf.

[80] Fortifyrights (2020). Myanmar: lift Internet restrictions to protect public health. Fortify Rights.

[81] Minority Rights: International Standards and Guidance for Implementation”, [Online]. Available: https://www.ohchr.org/sites/default/files/Documents/Publications/MinorityRights_en.pdf.

[82] Factsheet on The Citizenship (Amendment) Act in India | USCIRF,” 1700:37:10 2023. https://www.uscirf.gov/resources/factsheet-citizenship-amendment-act-india (accessed Mar. 17, 2023).

[83] Bajoria J. (2020). https://www.hrw.org/report/2020/04/10/shoot-traitors/discrimination-against-muslims-under-indias-new-citizenship-policy.

[84] Awaad R. (2021). Suicide attempts of Muslims compared with other religious groups in the US. JAMA Psychiatr..

[85] Staff N. (2018). http://publicintegrity.org/politics/as-intolerance-grows-targeted-religious-groups-join-forces/.

[86] An overview of federal higher education relief. https://lao.ca.gov/Publications/Report/4225.

[87] (2021). 1.1: Introduction to Multicultural Education.

[88] The Curb-Cut Effect (SSIR).” https://ssir.org/articles/entry/the_curb_cut_effect (accessed Apr. 25, 2023).

[89] Fighting misinformation in the time of COVID-19, one click at a time.” https://www.who.int/news-room/feature-stories/detail/fighting-misinformation-in-the-time-of-covid-19-one-click-at-a-time (accessed Mar. 17, 2023).

[90] Masland M.C., Lou C., Snowden L. (2010). Use of communication technologies to cost-effectively increase the availability of interpretation services in healthcare settings. Telemed. J. e Health.

